# Unveiling miR‐451a and miR‐142‐3p as prognostic markers in non‐small cell lung cancer via small extracellular vesicle liquid biopsy

**DOI:** 10.1002/ctm2.70634

**Published:** 2026-03-04

**Authors:** Miranda Burdiel, Ana Arauzo‐Cabrera, Julia Jiménez, Rocío Moreno‐Velasco, Carlos Rodríguez‐Antolín, Olga Pernía, Eva Madrid‐Cardenas, Oliver Higuera, Laura Gutiérrez‐Sainz, Paloma Yubero, Julia Villamayor, Itsaso Losantos‐García, Nadina Erill Sagalés, Víctor González Rumayor, Javier de Castro, Inmaculada de Ibáñez de Cáceres, Olga Vera

**Affiliations:** ^1^ Experimental Therapies and Biomarkers in Cancer IdiPAZ Madrid Spain; ^2^ Cancer Epigenetics Laboratory Clinical and Molecular Genetics Department (INGEMM) La Paz University Hospital Madrid Spain; ^3^ CIBERONC, ISCIII Madrid Spain; ^4^ Bioinformatics Unit Clinical and Molecular Genetics Department (INGEMM) La Paz University Hospital Madrid Spain; ^5^ Medical Oncology Department La Paz University Hospital Madrid Spain; ^6^ Biostatistics Unit La Paz University Hospital, IdiPAZ Madrid Spain; ^7^ Atrys Health Barcelona Spain

1

Dear Editor,

In our search for non‐invasive cancer biomarkers, we analysed the microRNome of small extracellular vesicles (sEVs) from cisplatin‐resistant and ‐sensitive cells. We found that elevated sEV‐miR‐451a and miR‐142‐3p predict poor prognosis in non‐small cell lung carcinoma (NSCLC). NSCLC is one of the most prevalent cancers worldwide and the deadliest.^1^ For advanced disease, current care combines platinum‐based chemotherapy with novel immunotherapies.^2^ However, many patients experience therapeutic failure due to innate or acquired resistance.

sEVs are small‐sized vesicles (30–150 nm) that represent a valuable source of tumour information and promising non‐invasive biomarkers.^3^ First, we co‐cultured sEVs previously isolated and characterised (see Supplementary Materials and Methods) from H23, A2780 and 41M cisplatin‐resistant cells (R‐sEVs)^4^ with their cisplatin‐sensitive counterparts. R‐sEVs uptake by sensitive cells was confirmed by flow cytometry (Figures 1A and S1). Then, sensitive (S), sensitive with R‐sEVS (S+R‐sEVs) and resistant (R) cells were exposed to their respective cisplatin Half‐maximal inhibitory concentration (IC50) dose.^5^ R‐sEVs increased cisplatin resistance in sensitive cells, surpassing the viability of the resistant cells (Figure 1B). High‐throughput miRNome sequencing of R‐sEVs and S‐sEVs followed by stringent bioinformatics analysis (Figure 1C) identified three miRNAs enriched in R‐sEVs: miR‐451, miR‐142‐3p (Table S1) and a novel miRNA, miR‐55745 (Table S2). quantitative Real‐Time PCR (Polymerase Chain Reaction) (qRT‐PCR) validation confirmed significant overrepresentation of miR‐451 and miR‐55745 in R‐sEVs across all cell lines (Figure 1D–F), whereas miR‐142‐3p was enriched in sEVs from H23R versus H23S and A2780R versus A2780S (Figure 1D,E). Intracellular analysis revealed that only miR‐142‐3p levels were significantly increased in H23R compared with H23S (*p* < .01) (Figure 1G–I).

**FIGURE 1:**
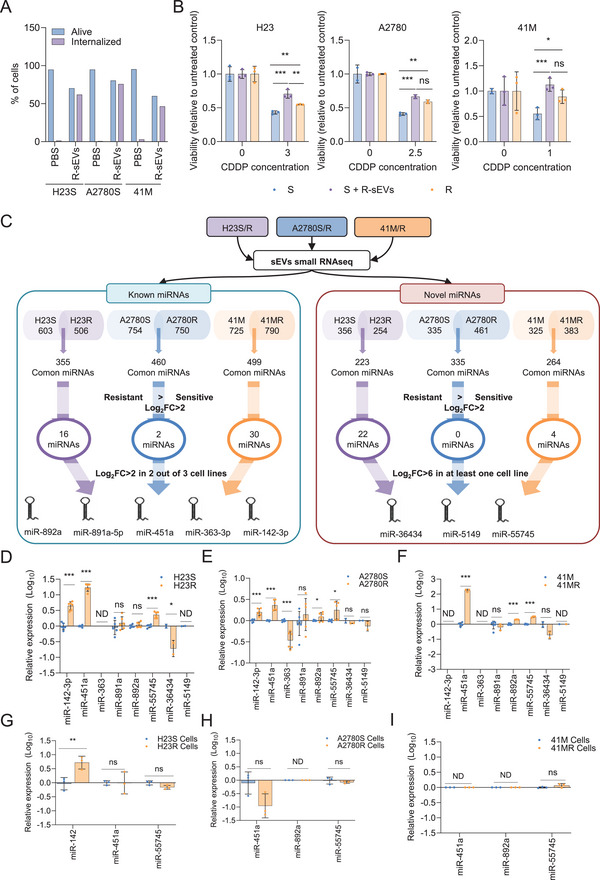
sEVs from CDDP‐resistant cells confer resistance to CDDP‐sensitive counterparts. (A) Quantification of sEVs internalisation from Figure S1. (B) Cell viability assay of sensitive (S), sensitive with resistant‐derived sEVs (S+R‐sEVs) and resistant (R) cells after exposure to CDDP for 48 h. One representative experiment out of two is shown. (C) Pipeline for the identification of cisplatin‐resistance sEV‐miRNAs. Total RNA isolated from sEVs collected from cisplatin‐sensitive (S) and ‐resistant (R) H23, A2780 and 41M were subjected to small‐RNA sequencing for the characterisation of known (left) and novel (right) miRNAs as potential markers of cisplatin resistance. FDR and Log2FC were considered to select eight miRNA candidates for further validation. Data are available at GSE204944. (D–F) qRT‐PCR analysis of the eight candidate miRNAs in the sEVs from the cisplatin‐sensitive (S) and ‐resistant (R) H23 (D), A2780 (E) and 41M (F). (G–I) qRT‐PCR analysis of miR‐451a, ‐142 and ‐55745 as cisplatin‐resistance candidate miRNAs in cisplatin‐sensitive (S) and ‐resistant (R) H23 (G), A2780 (H) and 41M (I). One representative experiment out of two is shown.*, *p* < .05; **, *p* < .01; ***, *p* < .001; ns, not significant; ND, not detected.

We then evaluated the levels of these miRNAs and an endogenous control^4^ in 127 pre‐treatment plasma samples from advanced NSCLC (stages III–IV). After that, all patients received standard treatment, either platinum‐based chemotherapy (CT; *n* = 78) or chemo‐immunotherapy (CT‐ICB; *n* = 49). Both cohorts were standardised by histology, sex and comorbidities, with stage III enriched in the CT cohort, and stage IV in the CT‐ICB cohort (Table S3), consistent with current management recommendations.^2^ Comparison of miRNA levels between NSCLC and control plasma samples (Figure 2) revealed significantly lower miR‐55745 levels in controls (Figure 2C), with no differences for miR‐451a or miR142‐3p (Figure 2A,B). Multivariate Cox analysis adjusted for stage, histology, smoking, sex, treatment and chronic obstructive pulmonary disease identified miR‐142‐3p (HR = 1.896, *p* = .017) and miR‐55745 (HR = 2.035, *p* = .033) as independent predictors of poor overall survival (OS). Tumour stage was the strongest predictor of progression‐free survival (PFS) (HR = 2.221, *p* = .004) while miR‐451a showed a non‐significant trend (HR = 1.642, *p* = .065). Stratification using optimal cut‐offs (Table S4) confirmed that high levels of all three miRNAs were significantly associated with NSCLC compared with healthy donors, regardless of treatment (Figure 2D–I). Additionally, in the CT cohort, high miR‐451a levels correlated with histology (*p* = .014), and high miR‐142‐3p levels correlated with stage (*p* = .012) and COPD (*p* = .006) (Table S5). No significant clinicopathological associations were observed in the CT‐ICB cohort (Table S6).

**FIGURE 2:**
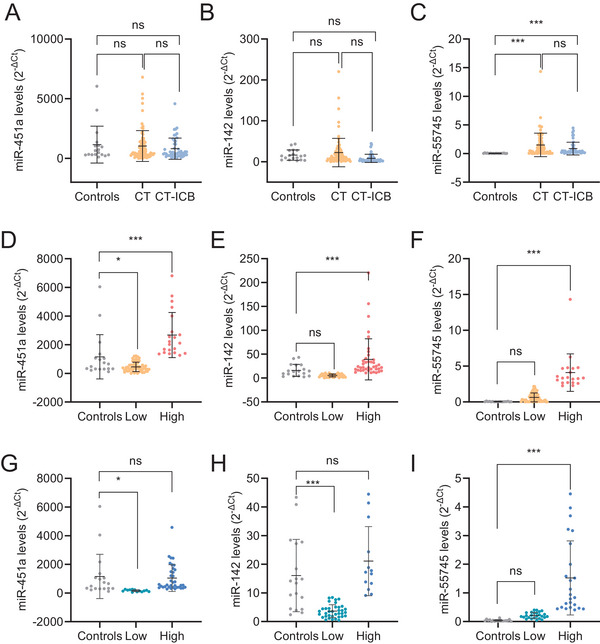
High levels of miR‐142‐3p, ‐451a and novel miR‐55745 differentiate lung cancer from control samples. (A–C) Basal levels of sEV‐miR‐451a (A), ‐miR‐142‐3p (B) and ‐miR‐55745 (C) measured by qRT‐PCR comparing healthy controls (*n* = 18) and 127 NSCLC patients, separated according to their follow‐up treatment (CT, chemotherapy; *n* = 78; CT‐ICB, chemo‐immunotherapy; *n* = 49). (D–F) Levels of sEV‐miR‐451a (D), ‐miR‐142‐3p (E) and ‐miR‐55745 (F) measured by qRT‐PCR comparing healthy controls (*n* = 18) and CT‐treated NSCLC patients segregated into ‘low’ and ‘high’ levels according to the 75th percentile (miR‐451a) or 50th percentile (miR‐142‐3p and miR‐55745). (G–I) Levels of sEV‐miR‐451a (G), ‐miR‐142‐3p (H) and ‐miR‐55745 (I) measured by qRT‐PCR comparing healthy controls (*n* = 18) and CT‐ICB‐treated NSCLC patients segregated into ‘low’ and ‘high’ levels according to the 25th percentile for miR‐451a, 75th percentile for miR‐142‐3p or 50th percentile for miR‐5574575. *, *p* < .05; ***, *p* < .001; ns, not significant.

Kaplan–Meier analysis linked high sEV‐miR‐451a, a known tumour suppressor associated with drug resistance,^6^ to poorer PFS and OS in both cohorts (Figure 3A–D). In the CT group, high miR‐451a increased progression risk by 2.196‐fold (*p* = .024) and death by 94.7% (*p* = .026) (Table S7); in the CT‐ICB cohort, relapse and death risk increased 4.163 (*p* = .009) and 2.886‐fold (*p* = .029), respectively (Table S8), consistent with its reported immunomodulatory role.^7^ Moreover, high miR‑451a levels in stage III patients from the CT cohort showed poorer PFS (*p* = .004) and OS (*p* = .008) (Figure 3E,F), with no differences in stage IV patients (Figure S2A,B). In the CT cohort, high miR‐142‐3p levels associated with lower OS (Figure 3G) and a 2.031‐fold increased risk of death (*p* = .012; Table S7), with no differences in PFS (Figure S2C). In the CT‐ICB cohort, elevated miR‐142‐3p was associated with poorer PFS and OS (Figure 3H,I), increasing relapse and death risks by 2.252‐fold (*p* = .047) and 2.696‐fold (*p* = .007), respectively (Table S8), consistently with its role as a negative regulator of anti‐tumour immunity.^8^ High miR‐142‐3p levels in stage IV CT patients specifically associated with poorer OS (*p* = .021) (Figure 2J) with no differences in stage III (Figure S2D). While higher levels of miR‐55745 were linked to poorer OS in the CT cohort (Figure 3K), no association was found for PFS or either stage in CT (Figure S2E–G) or CT‐ICB patients (Figure S2H,I). Nevertheless, its differential expression between NSCLC and controls and its unexplored role in lung cancer, its potential relevance, particularly in early stages, cannot be discounted. In the CT‐ICB cohort, stage‐specific analyses (IV and III/IV‐combined) yielded similar results (Figure S2J–O), likely reflecting very limited number of stage III cases (*n* = 8).

**FIGURE 3:**
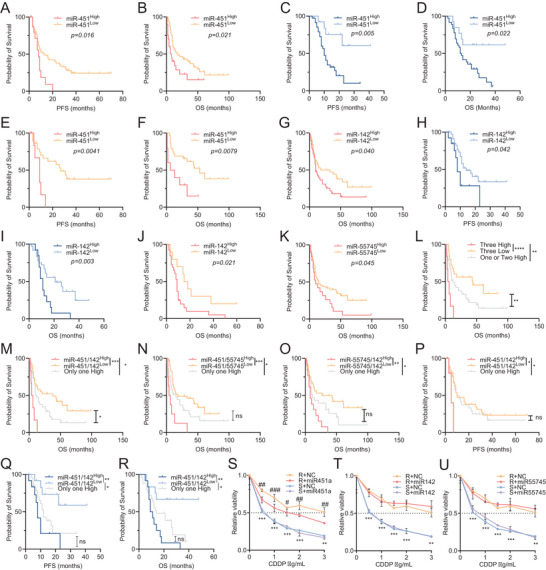
miR‐142‐3p and miR‐451a are key predictors of chemo‐immunotherapy resistance in advanced NSCLC. (A and B) Kaplan–Meier survival analysis comparing low (*n* = 58) and high (*n* = 20) miR‐451a levels in NSCLC patients treated with chemotherapy in terms of progression‐free survival (A) and overall survival (B) in months. (C and D) Kaplan–Meier survival analysis of low (*n* = 14) and high (*n* = 35) levels of miR‐451a in NSCLC patients treated with chemo‐immunotherapy in terms of progression‐free survival (C) and overall survival (D). (E and F) Kaplan–Meier survival analysis of low (*n* = 29) and high (*n* = 10) miR‐451a levels in stage III NSCLC patients treated with chemotherapy in terms of progression‐free survival (E) and overall survival (F). (G) Kaplan–Meier survival analysis of low (*n* = 40) and high (*n* = 38) levels of miR‐142‐3p in NSCLC patients treated with chemotherapy in terms of overall survival. (H and I) Kaplan–Meier survival analysis of low (*n* = 36) and high (*n* = 13) levels of miR‐142‐3p in NSCLC patients treated with chemo‐immunotherapy in terms of progression‐free survival (H) and overall survival (I). (J) Kaplan–Meier survival analysis of low (*n* = 11) and high (*n* = 28) miR‐142‐3p levels in stage IV NSCLC patients treated with chemotherapy in terms of overall survival. (K) Kaplan–Meier survival analysis of low (*n* = 59) and high (*n* = 19) levels of miR‐55745 in NSCLC patients treated with chemotherapy in terms of overall survival. (L) Kaplan–Meier survival analysis of low (*n* = 43), high (*n* = 6) and ‘one or two high’ (*n* = 29) levels of a combination of miR‐451a, ‐142‐3p and ‐55745 in NSCLC patients treated with chemotherapy in terms of overall survival. (M) Kaplan–Meier survival analysis of low (*n* = 34), high (*n* = 13) and ‘only one high’ (*n* = 31) levels of a combination of miR‐451a and ‐142 in NSCLC patients treated with chemotherapy in terms of overall survival. (N) Kaplan–Meier survival analysis of low (*n* = 44), high (*n* = 9) and ‘only one high’ (*n* = 25) levels of a combination of miR‐451a and ‐55745 in NSCLC patients treated with chemotherapy in terms of overall survival. (O) Kaplan–Meier survival analysis of low (*n* = 36), high (*n* = 14) and ‘only one high’ (*n* = 28) levels of a combination of miR‐142‐3p and ‐55745 in NSCLC patients treated with chemotherapy in terms of overall survival. (P) Kaplan–Meier survival analysis of low (*n* = 34), high (*n* = 13) and ‘only one high’ (*n* = 31) levels of a combination of miR‐451a and ‐142‐3p in NSCLC patients treated with chemotherapy in terms of progression‐free survival. (Q and R) Kaplan–Meier survival analysis of low (*n* = 12), high (*n* = 16) and ‘only one high’ (*n* = 12) levels of a combination of miR‐451 and ‐142‐3p in NSCLC patients treated with chemo‐immunotherapy in terms of progression‐free survival (Q) and overall survival (R). Log‐rank (Mantel–Cox) test was used for comparisons and *p* < .05 was considered as a significant change in OS or PFS. *, *p* < .05; **, *p* < .01; ***, *p* < .001; ****, *p* < .0001. (S–U) Effect of overexpression of miR‐451a (S), ‐142‐3p (T) or ‐55745 (U) on cell sensitivity to CDDP in H23 cell line. Viability curves of H23 transfected with negative control mimic (S+NC and R+NC) and with the overexpression mimics (S+miR‐451a; R+miR‐451a; S+miR‐142‐3p; R+miR‐142‐3p; S+miR‐55745; R+miR‐55745). Each experimental group was exposed for 48 h to six different CDDP concentrations, and data were normalised to each untreated control, set to 1. The data represent the mean ± SD of at least two independent experiments performed in triplicate at each drug concentration for each group analysed. R versus S: **, *p* < .01; ***, *p* < .001; R+NC vs R+miRNA: #, *p* < .05; ##, *p* < .01; ###, *p* < .001.

Last, we examined the combined miRNA effects on OS and PFS. High levels of all three miRNAs in the CT cohort (Figure 3L) associated with shorter OS and a 5.701‐fold death risk (*p* = .0001; Table S7) with a trend towards lower PFS (Figure S2P). High levels of combined miR‐451a/miR‐142‐3p, miR‐451a/miR‐55745 or miR‐142‐3p/miR‐55745 (Figure 3M–O) were associated with lower OS and increased death risk of 2.773 (*p* = .0001), 4.015 (*p* = .001) and 5.864 (*p* = .003), respectively (Table S7). High levels of combined miR‐451a/miR‐142‐3p also predicted lower PFS compared with both low (*p* = .049) or only‐one‐high (*p* = .047) (Figure 3P). While these results are promising, some subgroups contained few patients and should be interpreted cautiously and validated in larger cohorts. No significant PFS associations were observed for pairs involving miR‑55745 (Figure S2Q,R and Table S7). Similarly, in the CT‐ICB cohort, high levels of both miR‐142‐3p/miR‐451a were associated with poorer PFS and OS (Figure 3Q,R) and increased the risk of relapse or death in 5.832 (*p* = .004) and 5.512 times (*p* = .004) (Table S8). Notably, selective export of growth‐inhibitory miRNAs (especially miR‐451a, whose EV cargo may not reflect intracellular levels) could relieve their suppressive effects on proliferation and survival pathways, thereby promoting tumour growth and drug resistance.^9^, ^10^ Functionally, miR‐451a overexpression partially restored the cisplatin sensitivity in H23R (Figures 3H and S3A), with no effects for miR‐142‐3p or miR‐55745 (Figures 3T,U and S3B,C). This is consistent with the notion that prognostic sEV‑miRNAs can act through systemic or microenvironment‑dependent mechanisms not captured in monoculture assays.

Altogether, our study indicates that plasma‐derived sEV miR‐451a and miR‐142‐3p, individually and in combination, may serve as promising candidate biomarkers in liquid biopsy for advanced NSCLC patients treated with CT or CT‐ICB.

## AUTHOR CONTRIBUTIONS

*Conceptualisation*: MB, JJ and IIC. *Methodology*: MB, AA, JJ, RM, CRA and OP. Formal analysis: MB, AA, JJ, RM, CRA, OP, ILG, IIC and OV. *Investigation*: MB, AA, JJ, RM, CRA, OP, EMC, OH, LGS, PY, JV, ILG, NES, VGR, JDC, IIC and OV. *Resources*: OH, LGS, PY, JV, ILG, NES, VGR, JDC, IIC and OV. Writing original draft: JJ, OV and IIC. *Writing, review and editing*: MB, AA, JJ, RM, CRA, OP, EMC, OH, LGS, PY, JV, ILG, NES, VGR, JDC, IIC and OV. *Supervision*: OV and IIC. *Project administration*: OV and IIC. *Funding acquisition*: NES, VGR, JDC, IIC and OV. We declare that all the authors of this study have directly participated in the planning, execution or analysis of the study, and all the authors have read and approved the final version submitted, adhering to the guidelines of the ICMJE.

## CONFLICT OF INTEREST STATEMENT

The authors declare no conflicts of interest. The information provided in this study is included in a patent application process (EP2020/069659) and therefore it must be treated, solely and exclusively, based on the purposes of this paper, and should not be published if it does not respond to the purpose thereof. This application and its contents are protected by the Spanish Law on Intellectual and Industrial Property, prohibiting the distribution, reproduction, disclosure, transformation and sale of the entire document or part thereof, as well as the use, under any circumstances, of the trademarks appearing therein, without the prior express written consent of the Foundation for Biomedical Research of La Paz University Hospital‐IdiPAZ (FIBHULP), which holds the ownership.

## FUNDING INFORMATION

Instituto de Salud Carlos III and the European Regional Development Fund/European Social Fund FIS [ERDF/ESF], Una Manera de Hacer Europa, under grants: PI21/00145, PI24/00291. HR from ISCIII: CD22/00040; CM23/00159; CP24/00005 and MICIU/AEI/ 10.13039/501100011033 and by the ‘European Union NextGenerationEU/PRTR’ under grant CPP2022‐009545. This work was also supported by Caixa‐Impulse Validate program under CI20‐00182. This work was also supported by Fundacion Mutua Madrilena AP180852022.

## Supporting information



Supporting information

Supporting information

Supporting information

## Data Availability

The datasets generated and/or analysed during the current study are available in the GEO repository, number GSE204944.
