# Supplementary material for: Unveiling miR‐451a and miR‐142‐3p as prognostic markers in non‐small cell lung cancer via small extracellular vesicle liquid biopsy
Source: Clin Transl Med. 2026 Mar 4;16(3):e70634. doi: 10.1002/ctm2.70634 (PMC12960053; doi:10.1002/ctm2.70634)
Supplement: Supplementary file 1 — Supporting information [file CTM2-16-e70634-s002.docx]

**SUPPLEMENTARY MATERIALS AND METHODS**

**Cell culture**

The Lung and ovarian cancer cell lines H23 (NCI-H23) and A2780 were purchased from either the ATCC (Manassas, VA) or the ECACC (Sigma-Aldrich, Spain). All were maintained in RPMI supplemented with 10% exosome-depleted FBS. The FBS was depleted of bovine exosomes by ultracentrifugation at 100,000×*g* for 16 hours at 4°C. The CDDP-resistant variants, H23R and A2780R, were previously established by exposing the cells to increasing doses of the platinum-based drug^1,2^. The cisplatin-sensitive and -resistant ovarian cancer cell lines 41M and 41MR were provided by Dr Kelland (UK) and maintained in DMEM supplemented with 10% exosome-depleted FBS. All cell lines were routinely tested for mycoplasma by the IdiPAZ Cell Culture Facility and were STR -authenticated at the Genomics Core Facility (IIBm, CSIC-UAM).

**Clinical samples and data collection**

Plasma samples from 78 non-small cell lung cancer (NSCLC) patients diagnosed with locally advanced and advanced stages (stages IIIA to IV) between 2015 and 2021 at La Paz University Hospital were collected before they received any platinum-based treatment. Additionally, 49 plasma samples were collected from NSCLC patients diagnosed with locally advanced or advanced stages (stages IIIA to IV) between 2019 and 2022, prior to receiving platinum-based chemotherapy combined with immunotherapy. Follow-up was conducted according to the medical oncology division's criteria at La Paz University Hospital. We also collected plasma samples from 18 healthy donors to comprise the control cohort. All samples were processed in accordance with standard operating procedures and with the appropriate approval of the Human Research Ethics Committees, including informed consent within the context of research. All samples were processed within 30 minutes of collection using Vacutainer EDTA blood collection tubes; hemolyzed samples were discarded as recommended³. An independent observer recorded Clinical, pathological and therapeutic data, which was then blinded for statistical analysis. In brief, patients diagnosed with stages III–IV non-small cell lung cancer at University Hospital la Paz between 2015 and 2022 were eligible for inclusion in the study and were assigned to the “chemotherapy (CT)” or “Chemo-immunotherapy (CT-ICB)” cohort according to their treatment. Patients with driver mutations (EGFR, ROS, ALK) who were treated with targeted therapies or drugs other than platinum-based chemotherapy were excluded from the study. Subject demographics are summarised in **Table S1**. Our study examined sex as a biological variable and found no differences.

**Exosome labelling and uptake assays by flow cytometry**.

Small extracellular vesicles (sEVs) were isolated and characterised in accordance with the MISEV guidelines, as detailed in our previous publication. In brief, exosomes obtained by ultracentrifugation from cisplatin-resistant cell lines were validated by TEM, NTA and Western blot analysis of positive and negative markers. The sEV preparations used in the present study, for both the RNA-seq dataset reported previously and the in vitro co-culture assays included here, originate from the same initial, fully characterised experimental batch. To avoid duplicating published figures, we refer readers to our prior work, where the complete sEV characterisation dataset for these samples is provided^3^. Exosomes were fluorescently labelled using a PKH26 Red Fluorescent Cell Linker Mini Kit (Merck, Germany), following the manufacturer's protocol. Briefly, 250 µl of Dilutent C was mixed with 1 µl of PKH26 for each sample. The Exosome pellets were then mixed with the stain solution and incubated for four minutes. The labelling reaction was stopped by adding an equal volume of 3% BSA, 0.2 µm filtered 1x PBS. Labelled exosomes were then washed in 35 ml of 3% BSA, 0.2 µm filtered, 1x PBS, collected by ultracentrifugation at 100,000×*g* for two hours, and resuspended in PBS. The exact same process was performed with PBS as a control to determine the PKH26 background.

For the uptake assays, sensitive cells were labelled with CellTrace Violet (CTV) (Thermofisher Scientific, USA). Briefly, 10⁶ cells resuspended in PBS + 5% exosome-depleted FBS were incubated for 20 minutes with 20 µL of a 1:100 dilution of CTV. After washing off the excess dye with culture medium, the cells were co-seeded with exosomes or PBS labelled with PKH26. After 20 hours of co-incubation, the cells were trypsinised, washed, and passed through the FACS Canto II flow cytometer (BD Biosciences, USA). To measure the death rate associated with incubation with exosomes, cells were stained with 7-Aminoactinomycin D (7AAD) (BD Biosciences, USA). The Results were analysed using FlowJo (FLOWJO LLC, USA).

**sEV functional assays**

H23, A2780 and 41M sensitive cells were plated in 96-well plates at a concentration of 20,000 cells per well. sEVs isolated from resistant cells were quantified using a Bradford assay (Bio-rad, California, USA) and co-incubated with sensitive cells at a concentration of 80 µg/ml for 48 hours. This was followed by treatment with the respective ResistantIC_50_ dose of cisplatin (Farmaterra, Spain): 3.0 µg/ml for H23, 2.5 µg/ml for A2780 and 1.0 µg/ml for 41M. Cell viability was measured 72 hours after drug treatment using the CellTiter 96® AQueous One Solution Cell Proliferation Assay (MTS) (Promega, Spain). PBS-treated cells were used as a control.

**Small RNAseq**
Cisplatin-sensitive/resistant paired H23S/H23R, A2780S/A2780R and 41S/41R small extracellular vesicle (sEV) preparations were used for the Small-RNAseq. RNA was extracted using a miRCURY RNA Isolation Kit – Cell and Plant (Exiqon, Denmark), following the manufacturer's instructions. The RNA was quantified using a NanoDrop ND-1000 spectrophotometer (Thermo Fisher Scientific, USA) and a Qubit 4 fluorometer (Invitrogen, Thermo Fisher Scientific, USA), and then analysed using Arraystar (Arraystar Inc., USA). Total RNA from each sample was used to prepare the miRNA sequencing library. 3' and 5' adapter ligation, cDNA synthesis, PCR amplification, and size selection between 130 and 150 bp (corresponding to 15–35 nucleotides of miRNAs) were performed. The DNA was sequenced with 51 cycles using an Illumina HiSeq 2000 sequencer (Illumina Inc., USA). The trimmed reads (length ≥ 15 nt and adapter removal) were aligned to human pre-miRNAs in miRBase 21^4^ using the NovoAlign software (NovoCraft, Malaysia) and the miRNA read counts were normalised as tag counts per million (TPM) of alignments. To select candidate miRNAs that were differentially represented between the sensitive and resistant phenotypes, reads with counts of less than two were discarded. Next, the remaining reads were normalised as transcripts per million aligned reads (TPM). To avoid false negatives, the miRNAs showing reads in both the sensitive and resistant subtypes were selected. For comparisons between phenotypes, the Log2 of Fold Change (log2FC) and p-value for each group were calculated. Then, pre-miRNAs with a log2FC greater than or equal to 2 were selected. Finally, the miRNAs that met these conditions in at least two of the three lines analysed were selected. Novel microRNAs are those that are not currently annotated in the miRBase database, but which can be identified using prediction algorithms based on counts obtained from massive miRNA sequencing. Novel miRNAs were predicted using the miRDeep2^5^ algorithm, which employs a probabilistic model of miRNA precursor processing by Dicer to report novel findings with high confidence. To enhance the sensitivity of the prediction, sequence data from all 3'-adapter trimmed files were pooled together. The prediction pipeline applied strict filtering criteria to ensure data quality: read Length (sequences shorter than 17 nucleotides were excluded from the analysis); alignment mismatches (reads were mapped to the reference genome, allowing a maximum of one mismatch); and candidate selection (putative novel miRNAs were identified based on the compatibility of their precursor secondary structure with Dicer processing). The same steps were followed for the selection of novel and known miRNAs, except for the final screening step, in which those with a log2FC greater than or equal to 6 in any line were selected. Data is available at GSE204944.

**qRT-PCR**

All miRNAs from each sample were non-specifically retrotranscribed using the TaqMan™ Advanced miRNA cDNA Synthesis Kit (Thermo Fisher Scientific, USA) according to the manufacturer's instructions. The same starting plasma volume was used for plasma sEVs and the same RNA input was used for cell-derived sEVs (i.e. 10 ng) across samples. Quantitative analysis of each specific miRNA was performed using TaqMan Advanced miRNA assays (hsa-miR-151a-3p: 477919_mir, hsa-miR-451a: 478107_mir and hsa-miR-451a: 477910_mir. Thermo Fisher Scientific designed a custom probe for hsa-miR-55745 using the following information: chromosome 4, coordinate 55745, precursor coordinate chr4:76846064-76846129 (hg38), and consensus mature sequence agugaaaugacuugagagg. TaqMan Universal PCR Master Mix (Thermo Fisher Scientific, USA) was used for qPCR amplification. All samples were analysed in triplicate using an HT7900 Real-Time PCR System thermocycler (Applied Biosystems, USA), with the following settings: 10 min at 95°C, followed by 40 cycles of 15 s at 95°C and 1 min at 60°C. Analysis of the results was performed using RQ Manager software (Thermo Fisher Scientific, USA), with relative miRNA levels calculated according to the comparative threshold cycle method 22^-ΔCt^, where ΔCt is determined by subtracting the Ct value of the endogenous control (miRNA-151a^3^) from the Ct value of the targeted miRNA.

**Cell transfection and microRNA (miRNA) functional assays**

Each cell line was plated in 24-well plates at a density of 40,000 cells per well and incubated for 24 hours. The cells were then transfected with 20nM of either miR-451a (Ref: MC10286), miR-142-3p (Ref: MC10398), or the negative control (Ref: 4464058) (all from Thermo Fisher Scientific, USA) using JetPrime (PolyPlus Transfection, France), following the manufacturer's protocol. The Mimic for miR-55745 was synthesised based on its mature sequence by Thermo Fisher Scientific (USA). After 6 hours of transfection, the cells were treated with increasing doses of cisplatin, as previously reported^2^. After 72 hours of treatment, the cells were fixed with 1% glutaraldehyde (Merck, Germany) and stained with 0.1% crystal violet (Merck, Germany). The Dye was then extracted using 10% acetic acid, and the absorbance was measured at 595 nm using an Infinite 200 PRO multimode reader (TECAN, Switzerland). To validate microRNA (miRNA) overexpression, 200,000 cells were plated in 6-well dishes and transfected with 20nM of each mimic for 72 hours, followed by RNA isolation and quantification by quantitative reverse transcription PCR (qRT-PCR) analysis.

**Statistical analysis**

The patients' clinical characteristics were described for the complete series using mean and standard deviation values or relative frequencies. The data were stratified according to whether patients showed high or low levels of the analysed miRNAs in plasma. All samples were collected at baseline, before any systemic therapy was started. Therefore, analyses assess the pretreatment prognostic association of miRNA levels with outcomes. Patients were subgrouped into “low” and “high” expression levels using the X-tile algorithm, which systematically evaluates all possible cut-off points in the expression distribution and identifies the one that best discriminates clinical outcomes, as previously described^6^. As a consequence of this data-driven approach, different miRNAs showed different optimal thresholds. For some miRNAs, the 50th percentile (median) maximised the log-rank test statistic, while for others, the 75th percentile provided the greatest separation between survival curves. This variability reflects biological and distributional differences among miRNAs. Following stratification, their distributions were compared using the chi-squared test or Fisher's exact test for qualitative variables and the Student's t -test or Wilcoxon–Mann–Whitney test for quantitative variables. Multivariate Cox proportional hazards models were used to analyse the association between miRNA levels and overall survival (OS) and progression-free survival (PFS). Covariates included tumour stage (III vs. IV), histology (adenocarcinoma vs. squamous), smoking status, sex, treatment type (CT vs. CT-ICB), the presence of COPD, and the expression levels of miR-451a, miR-142-3p and miR-55745 (categorised as high or low based on X-tile-derived cutoffs). Hazard ratios (HR), 95% confidence intervals and p-values are reported. OS and PFS were estimated using the Kaplan–Meier method and compared between groups using the log-rank test. All p-values were two-sided and the type I error rate was set at 5%. A p-value below 0.05 was considered statistically significant. In vitro experiments were performed in triplicate or quadruplicate, and each experiment was repeated at least twice. Unless otherwise indicated, one representative experiment is shown. Data represent the mean ± SD. All statistical analyses were performed using SAS 9.3 (SAS Institute, Cary, NC, USA) and RStudio (version 1.1.423). Values of p < 0.05 were considered statistically significant: * p < 0.05, ** p < 0.01, and *** p < 0.001.

**References**

1 Ibanez de Caceres, I. *et al.* IGFBP-3 hypermethylation-derived deficiency mediates cisplatin resistance in non-small-cell lung cancer. *Oncogene* **29**, 1681-1690, doi:10.1038/onc.2009.454 (2010).

2 Vera, O. *et al.* DNA Methylation of miR-7 is a Mechanism Involved in Platinum Response through MAFG Overexpression in Cancer Cells. *Theranostics* **7**, 4118-4134, doi:10.7150/thno.20112 (2017).

3 Burdiel, M. *et al.* MiR-151a: a robust endogenous control for normalizing small extracellular vesicle cargo in human cancer. *Biomarker research* **11**, 94, doi:10.1186/s40364-023-00526-0 (2023).

4 Kozomara, A. & Griffiths-Jones, S. miRBase: annotating high confidence microRNAs using deep sequencing data. *Nucleic Acids Res* **42**, D68-73, doi:10.1093/nar/gkt1181 (2014).

5 Friedlander, M. R., Mackowiak, S. D., Li, N., Chen, W. & Rajewsky, N. miRDeep2 accurately identifies known and hundreds of novel microRNA genes in seven animal clades. *Nucleic Acids Res* **40**, 37-52, doi:10.1093/nar/gkr688 (2012).

6 Camp, R. L., Dolled-Filhart, M. & Rimm, D. L. X-tile: a new bio-informatics tool for biomarker assessment and outcome-based cut-point optimization. *Clin Cancer Res* **10**, 7252-7259, doi:10.1158/1078-0432.CCR-04-0713 (2004).
