# Supplementary material for: Unveiling miR‐451a and miR‐142‐3p as prognostic markers in non‐small cell lung cancer via small extracellular vesicle liquid biopsy
Source: Clin Transl Med. 2026 Mar 4;16(3):e70634. doi: 10.1002/ctm2.70634 (PMC12960053; doi:10.1002/ctm2.70634)
Supplement: Supplementary file 3 — Supporting information [file CTM2-16-e70634-s001.pptx]

## Slide 1
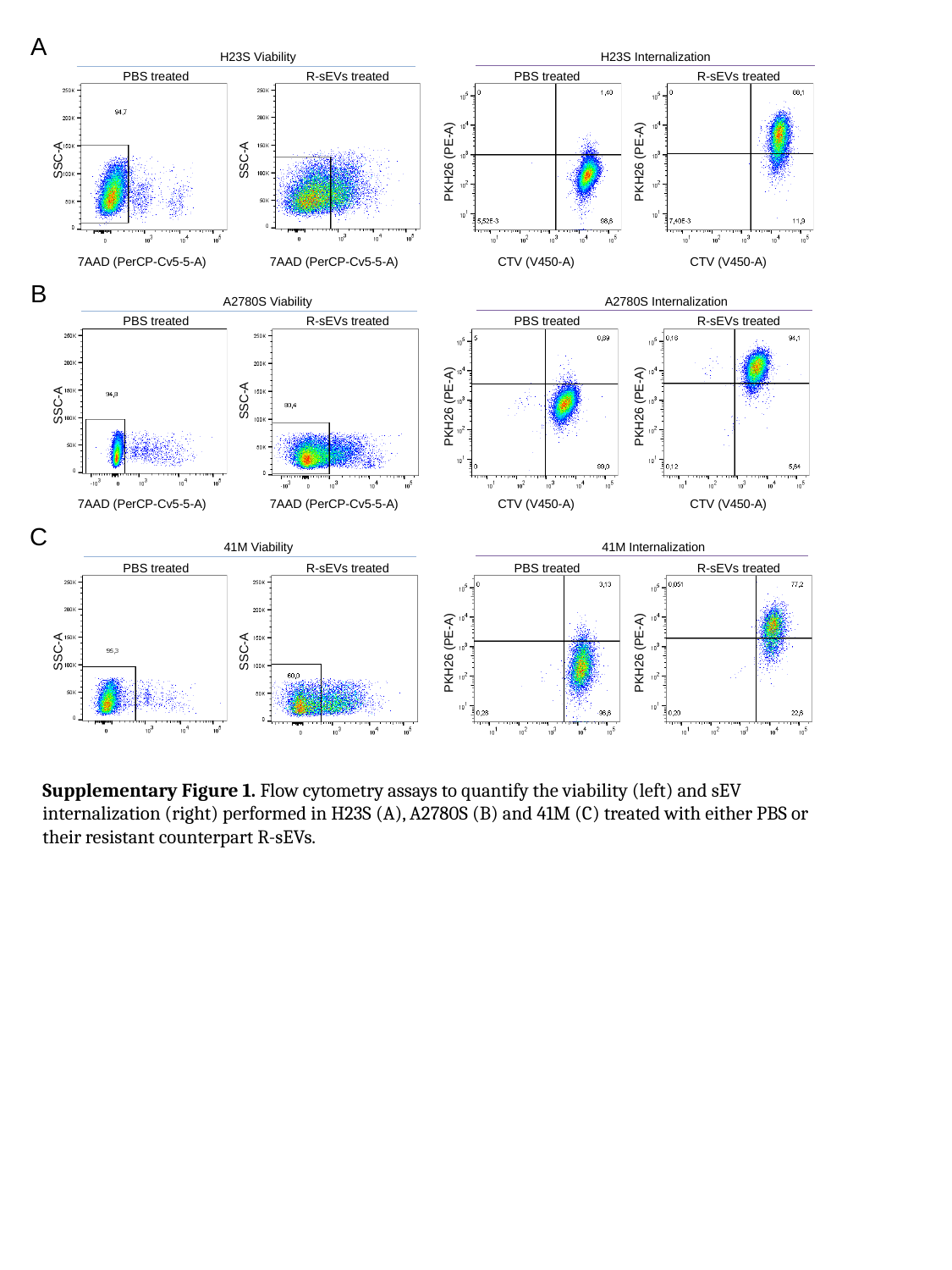

A
H23S Internalization
H23S Viability
PBS treated
R-sEVs treated
PBS treated
R-sEVs treated
 SSC-A
 SSC-A
PKH26 (PE-A)
PKH26 (PE-A)
7AAD (PerCP-Cv5-5-A)
7AAD (PerCP-Cv5-5-A)
CTV (V450-A)
CTV (V450-A)
B
A2780S Internalization
A2780S Viability
PBS treated
R-sEVs treated
PBS treated
R-sEVs treated
 SSC-A
PKH26 (PE-A)
 SSC-A
PKH26 (PE-A)
7AAD (PerCP-Cv5-5-A)
7AAD (PerCP-Cv5-5-A)
CTV (V450-A)
CTV (V450-A)
C
41M Internalization
41M Viability
PBS treated
R-sEVs treated
PBS treated
R-sEVs treated
 SSC-A
 SSC-A
PKH26 (PE-A)
PKH26 (PE-A)
Supplementary Figure 1. Flow cytometry assays to quantify the viability (left) and sEV internalization (right) performed in H23S (A), A2780S (B) and 41M (C) treated with either PBS or their resistant counterpart R-sEVs.

## Slide 2
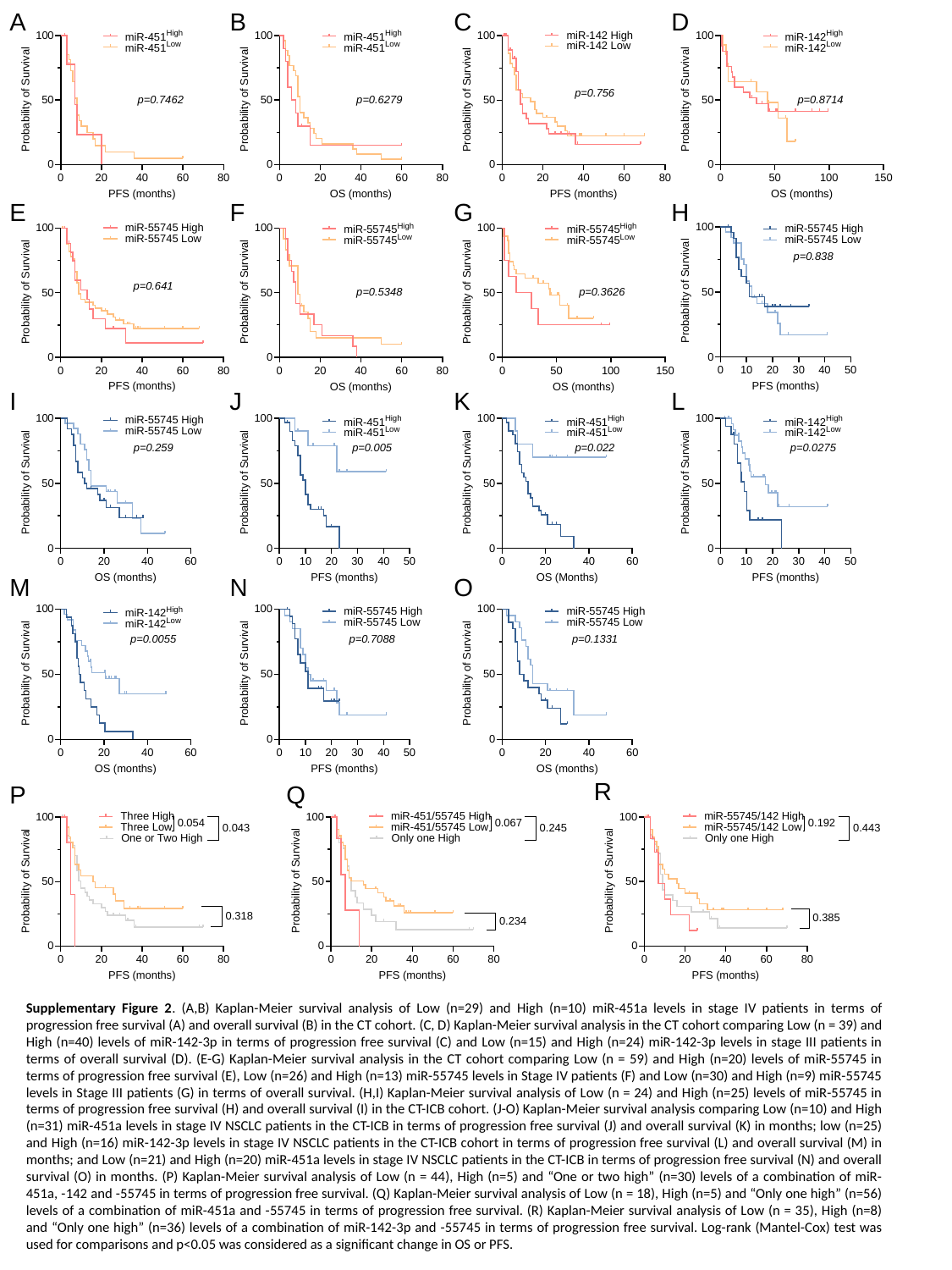

A
D
B
C
E
F
G
H
I
J
K
L
M
N
O
R
P
Q
Supplementary Figure 2. (A,B) Kaplan-Meier survival analysis of Low (n=29) and High (n=10) miR-451a levels in stage IV patients in terms of progression free survival (A) and overall survival (B) in the CT cohort. (C, D) Kaplan-Meier survival analysis in the CT cohort comparing Low (n = 39) and High (n=40) levels of miR-142-3p in terms of progression free survival (C) and Low (n=15) and High (n=24) miR-142-3p levels in stage III patients in terms of overall survival (D). (E-G) Kaplan-Meier survival analysis in the CT cohort comparing Low (n = 59) and High (n=20) levels of miR-55745 in terms of progression free survival (E), Low (n=26) and High (n=13) miR-55745 levels in Stage IV patients (F) and Low (n=30) and High (n=9) miR-55745 levels in Stage III patients (G) in terms of overall survival. (H,I) Kaplan-Meier survival analysis of Low (n = 24) and High (n=25) levels of miR-55745 in terms of progression free survival (H) and overall survival (I) in the CT-ICB cohort. (J-O) Kaplan-Meier survival analysis comparing Low (n=10) and High (n=31) miR-451a levels in stage IV NSCLC patients in the CT-ICB in terms of progression free survival (J) and overall survival (K) in months; low (n=25) and High (n=16) miR-142-3p levels in stage IV NSCLC patients in the CT-ICB cohort in terms of progression free survival (L) and overall survival (M) in months; and Low (n=21) and High (n=20) miR-451a levels in stage IV NSCLC patients in the CT-ICB in terms of progression free survival (N) and overall survival (O) in months. (P) Kaplan-Meier survival analysis of Low (n = 44), High (n=5) and “One or two high” (n=30) levels of a combination of miR-451a, -142 and -55745 in terms of progression free survival. (Q) Kaplan-Meier survival analysis of Low (n = 18), High (n=5) and “Only one high” (n=56) levels of a combination of miR-451a and -55745 in terms of progression free survival. (R) Kaplan-Meier survival analysis of Low (n = 35), High (n=8) and “Only one high” (n=36) levels of a combination of miR-142-3p and -55745 in terms of progression free survival. Log-rank (Mantel-Cox) test was used for comparisons and p<0.05 was considered as a significant change in OS or PFS.

## Slide 3
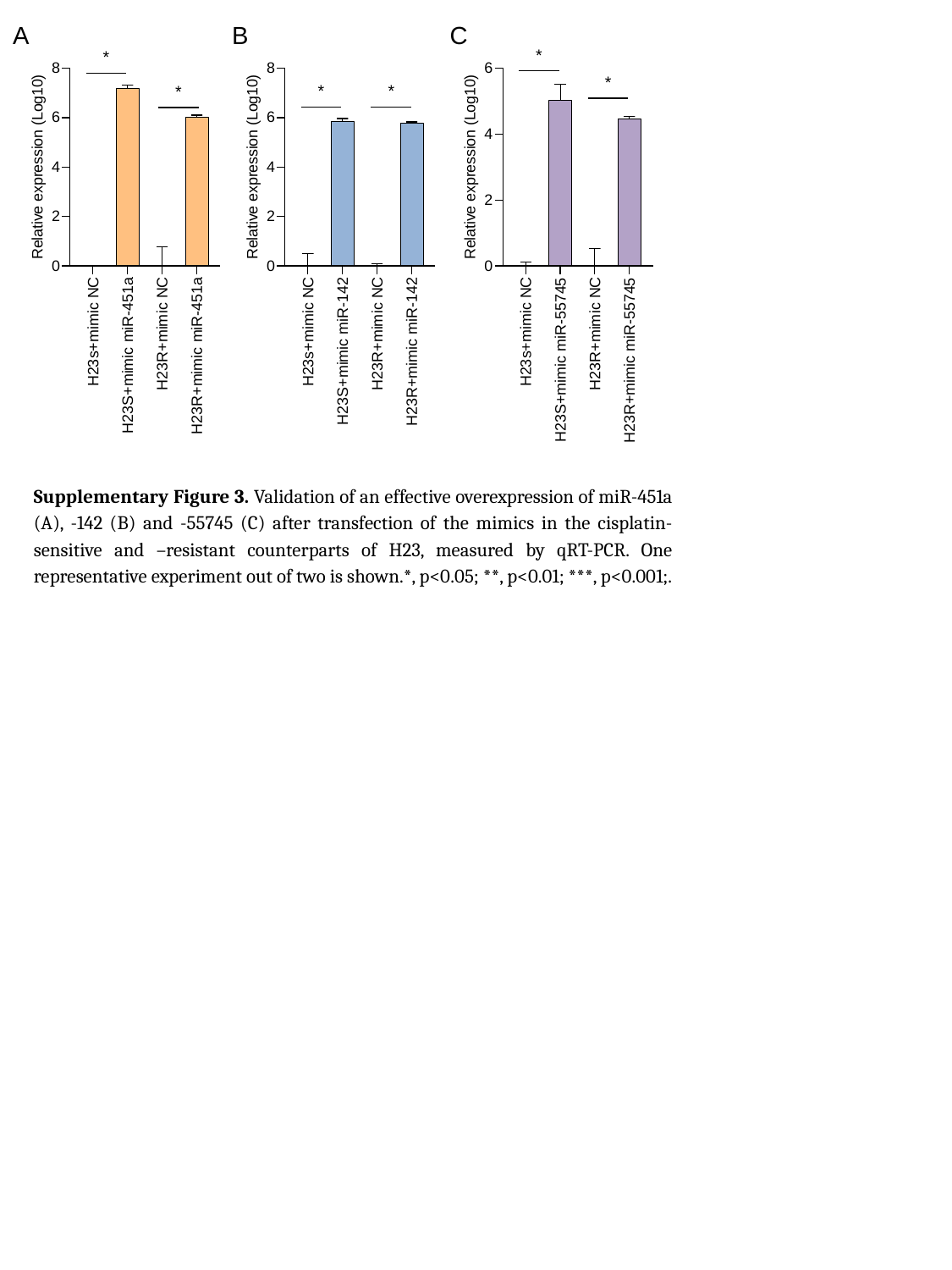

A
B
C
Supplementary Figure 3. Validation of an effective overexpression of miR-451a (A), -142 (B) and -55745 (C) after transfection of the mimics in the cisplatin-sensitive and –resistant counterparts of H23, measured by qRT-PCR. One representative experiment out of two is shown.*, p<0.05; **, p<0.01; ***, p<0.001;.
